# Age-related macular degeneration (AMD): an introduction

**Published:** 2025-01-31

**Authors:** David Yorston

**Affiliations:** 1 Consultant Ophthalmologist, Tennent Institute of Ophthalmology, Gartnavel Hospital, Glasgow, Scotland.

**AMD is a progressive disease of the macula, causing loss of central vision, and is more prevalent in older people.**

Worldwide, age-related macular degeneration (AMD) causes more visual impairment than any other condition other than refractive error and cataract. An analysis of prevalence studies suggests that there were 196 million people with AMD in 2020; this will increase to 288 million by 2040.

The reason for this increase is demographic change: as the proportion of the population aged over 65 increases, so will the prevalence of AMD, as it is a degenerative process and is more prevalent in older people.

AMD affects the **outer retinal complex** at the macula, consisting of the photoreceptors (also known as the rods and cones), the retinal pigment epithelium (RPE), Bruch's membrane, the choriocapillaris, and the choroid (Figure 1). Damage to one component of this complex rapidly affects all the other components. Once the photoreceptors at the macula are destroyed, central vision is lost.

**Figure 1:**
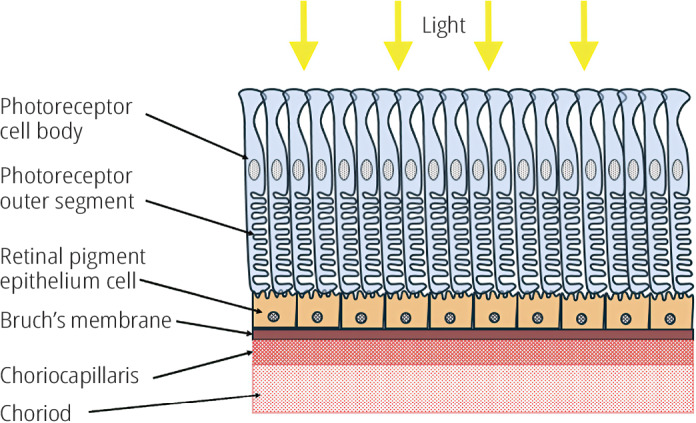
**Normal macula.** Cross-section of the outer retinal complex, showing the photoreceptors (cell body and outer segment), retinal pigment epithelium (RPE), Bruch's membrane, choriocapillaris, and choroid.

© DAVID YORSTON CC BY-NC-SA 4.0

In a healthy macula, the outer segments of the photoreceptors are continually being renewed. The oldest parts of the photoreceptor outer segments are broken down by the RPE and removed by the circulation in the choriocapillaris. As we age, this process becomes less efficient, and the half-digested remains of the photoreceptor outer segments build up in Bruch's membrane – the layer between the choriocapillaris and the retinal pigment epithelium. This is visible as small, pale sub-retinal spots, called drusen (Figure 2).

**Figure 2:**
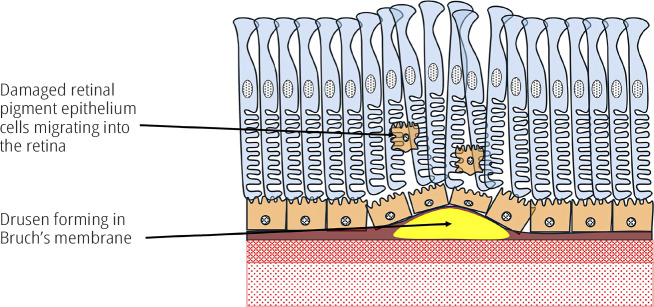
**Early AMD.** The retinal pigment epithelium (RPE) can no longer completely break down the older parts of the photoreceptor outer segments. The remains build up in the thickened Bruch's membrane, as drusen. Damaged RPE cells migrate into the retina and are visible as small black spots.

© DAVID YORSTON CC BY-NC-SA 4.0

A combination of chronic inflammation and reduced circulation in the choriocapillaris then leads to damage to the outer retinal complex, causing loss of vision. This can take the form of atrophy of the RPE and photoreceptors (atrophic or ‘dry’ AMD), or the development of abnormal new vessels under the retina (neovascular/exudative or ‘wet’ AMD). These two types often co-exist in the same patient, but it is clinically useful to distinguish between them.

**Atrophic (‘dry’) AMD** (Figure 4) is due to atrophy of the retinal pigment epithelium, which leads to loss of the photoreceptors. Loss of vision is usually slow and gradual, and is irreversible. When it affects the fovea, there will be severe visual impairment.

**Figure 3:**
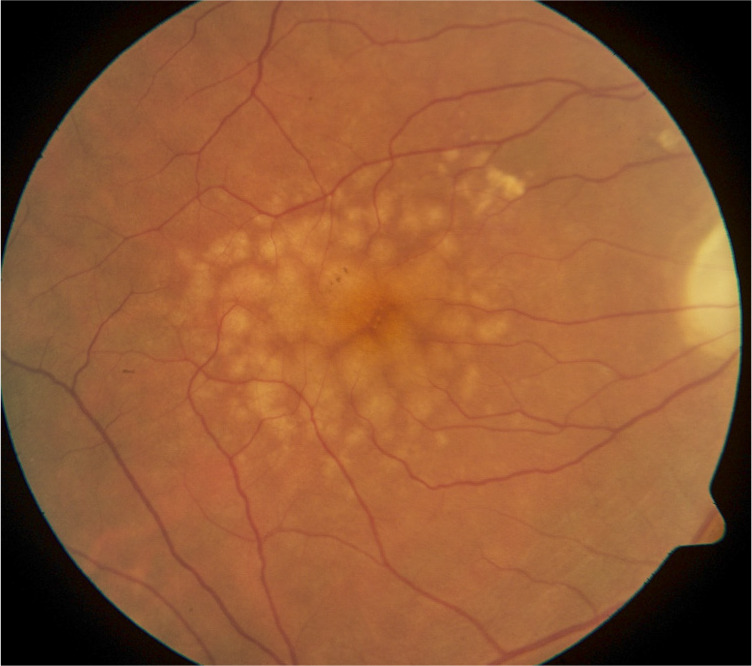
Drusen in early AMD, seen using an ophthalmoscope.

© IPOLIKER CC BY-NC-SA 4.0

**Figure 4:**
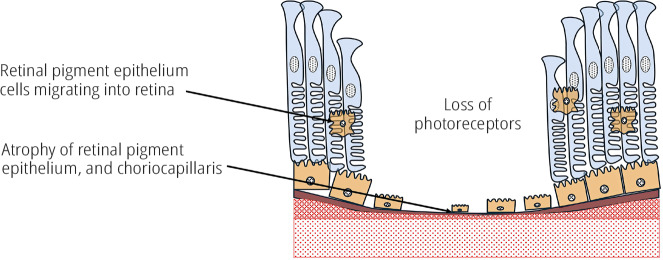
**Advanced AMD: atrophic (dry) type**. There is atrophy of the retinal pigment epithelium and choriocapillaris, with resulting loss of photoreceptors.

© DAVID YORSTON CC BY-NC-SA 4.0

**Neovascular (‘wet’ or exudative) AMD** (Figure 6) is the result of new, abnormal blood vessels growing under the RPE. These leak fluid into or under the retina, causing swelling (oedema). In the initial stages, vision may be distorted (metamorphopsia) because the oedema results in misalignment of the photoreceptors. Chronic leakage leads to exudates, and sometimes these new vessels rupture, leading to bleeding under the RPE or under the photoreceptor cells (sub-retinal bleeding). Loss of vision is usually more rapid, occurring over days or weeks, but can happen suddenly, particularly if there is sub-retinal bleeding.

**Figure 5:**
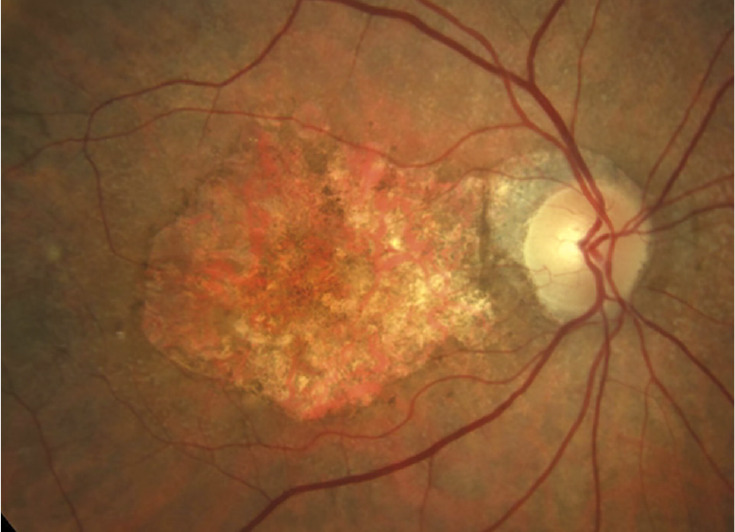
Atrophy (pale area) in a patient with dry AMD.

JORDI MONÉS BARCELONAMACULAFOUND.ORG CC BY-NC-ND 4.0

**Figure 6:**
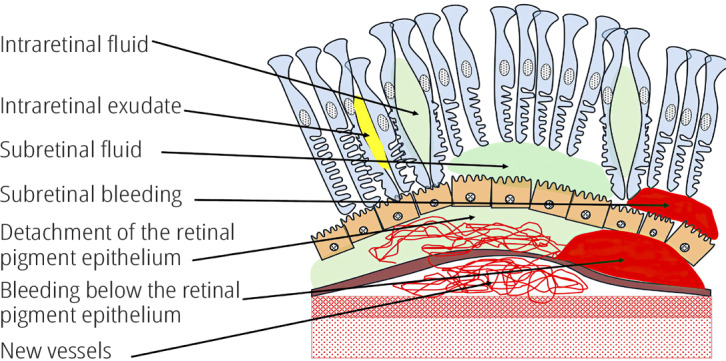
**Advanced AMD: neovascular (wet) type.** New vessels have grown from the choriocapillaris into the space under the retinal pigment epithelium. Note the distorted and misaligned photoreceptors, leading to metamorphopsia.

© DAVID YORSTON CC BY-NC-SA 4.0

## What causes AMD?

We know that certain **genetic abnormalities** increase the risk of developing AMD, particularly mutation in the gene for complement factor H, a protein involved in the regulation of inflammation. If complement H does not function normally, it is thought that inflammation caused by the inefficient removal of photoreceptor outer segments is worse; this leads to damage to the surrounding cells.

We also know that **cigarette smoking** greatly increases the risk of AMD. Tobacco smoke contains chemicals that promote inflammatory responses and increase the risk that early AMD will progress.

## Prevention

Reducing genetic risk is impossible, unless you can choose your parents! However, stopping smoking is valuable. Twenty years after quitting, an ex-smoker's risk of AMD is the same as that of a non-smoker. Dietary measures may help, but the evidence is unclear. A diet that is low in fats, but high in fish and vegetables, may reduce the risk of AMD. Regular exercise and avoiding obesity also seem to reduce the risk. A large clinical trial (AREDS – Age-Related Eye Disease Study) showed that daily high doses of antioxidants – specifically vitamin C (500 mg), vitamin E (400 IU), zinc (80 mg) and copper (2 mg) – reduced the risk of AMD progression by about 25% in patients who already had severe AMD in one eye. However, there was no beneficial effect in patients with early AMD. These doses of vitamins far exceed the normal dietary levels – for example, the recommended minimum daily intake of vitamin C is around 100 mg – and can only be achieved by taking tablets.

## Symptoms

The main symptom of AMD is loss of central vision. Unlike glaucoma, or diabetic retinopathy, where advanced disease may be asymptomatic, significant AMD will always cause reduced visual acuity in the affected eye.

In **atrophic AMD**, the loss of vision is likely to be slow and gradual, extending over months or years. Because the photoreceptors are not displaced, there are usually no symptoms of distortion.

In **neovascular AMD**, loss of vision is usually more rapid, occurring over days or weeks, and can happen suddenly – particularly if there is a haemorrhage under the retina. In the initial stages, when fluid leaks into or under the retina, the photoreceptors are displaced, causing distortion or metamorphopsia. This is an important symptom of macular disease but the patient may not think to mention it. You should always ask about distortion, and, if the patient is unsure, test for it by asking them to look at a straight line, such as a doorway or window frame. You don't need special charts or instruments to detect whether metamorphopsia is present. Quantifying metamorphopsia is more complex, and probably not essential in the management of AMD.

## Clinical features

The clinical features of advanced AMD are easily detected by examining the macula with an ophthalmoscope. However, the early changes can be more subtle.

**Figure 7:**
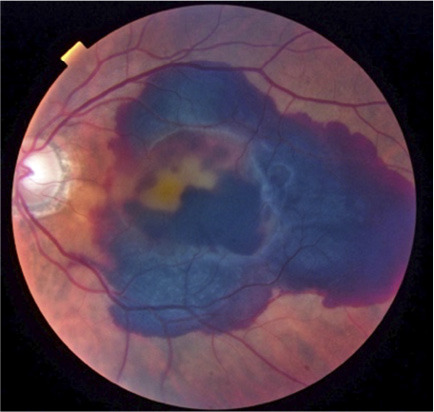
Submacular haemorrhage in advanced neovascular AMD. Note that the blood vessels are visible above the bleeding, confirming that it is subretinal.

The earliest visible sign of AMD is **drusen** (Figure 3). Small drusen are common and have no clinical significance. However larger drusen – 100 microns or more – are an indication that the outer retinal complex is damaged. There may be retinal pigment epithelium migration into the retina, visible as black spots. If the AMD is primarily atrophic, there will be **pale areas** or atrophy (Figure 5), caused by loss of RPE and choriocapillaris. If there are macular new vessels, you may see **oedema** and **intra-retinal or sub-retinal fluid**. If the vessels continue to leak, there are likely to be **exudates**, and there may be **bleeding under the retina** (Figure 7). Ultimately, the macula is replaced by white scar tissue.

© DAVID YORSTON CC BY-NC-SA 4.0

## Investigations

For atrophic AMD, very precise measurements of the extent of the atrophy can be obtained with autofluorescence. A photograph of the retina is taken using blue light (488 nm). This causes the lipofuscin in the RPE cells to fluoresce. Where there is atrophy of the RPE, it shows up as a dark area in the image. In practice, autofluorescence imaging is not necessary to make a diagnosis of atrophic AMD, but it may be useful for measuring progression.

The most useful investigation for AMD is optical coherence tomography (OCT). This is covered in more detail on pp. 15–16. Essentially, OCT uses low powered infrared lasers to create a detailed cross-sectional image of the retina. This demonstrates atrophy and loss of photoreceptors, and/or intra-retinal or sub-retinal fluid. OCT angiography (OCT-A) bounces the laser off moving red cells, and uses the signal this generates to create a detailed image of the retinal circulation, including any abnormal vessels growing from the choroid. OCT is safe and takes about five minutes per patient. This means that it can be repeated at each clinic visit, and used to follow the response to treatment or to indicate when treatment needs to be increased.

## Treatment

Anti-VEGF drugs (see page 10) are effective against neovascular AMD, but have no effect on atrophic AMD, which usually co-exists with neovascular AMD. Most patients with treated neovascular AMD will therefore still experience a gradual loss of visual acuity over several years. We must ensure that low vision and supportive services are available to these patients.

